# Small Mitral Valve Orifice Area in Mitral Valve Transcatheter Edge-to-Edge Repair: The REPAIR Study

**DOI:** 10.1016/j.jacadv.2026.103014

**Published:** 2026-06-25

**Authors:** Philipp von Stein, Jennifer von Stein, Florian Schindhelm, Felix Rudolph, Paula Sagmeister, Jean Marc Haurand, Jannik Jobst, Matthias Gröger, Lukas Stolz, Carl Schulz, Christoph Alexander Mues, Donika Mustafa, Isabel A. Hoerbrand, Atsushi Sugiura, Charlotte Wolff, Philipp Lurz, Philipp Jahn, Guido Ascione, Henning Guthoff, Stephan Baldus, Wolfgang Rottbauer, Muhammed Gerçek, Roman Pfister, Tienush Rassaf, Marcel Weber, Mathias Konstandin, Helge Möllmann, Niklas Schofer, Jörg Hausleiter, Mirjam Kessler, Bernhard Unsöld, Patrick Horn, Tobias Kister, Volker Rudolph, Amir Abbas Mahabadi, Juan F. Granada, Victor Mauri, REPAIR Investigators, Ralph-Stephan von Bardeleben, Cecilia Ennin, Kai Peter Friedrichs, Christina Grothusen, Malte Kelm, Georg Nickenig, Kerstin Piayda, Amin Polzin, Tobias Ruf, Leonhard Moritz Schneider, Holger Thiele, Fabian Voss, Laila Widmann

**Affiliations:** a Department of Cardiology, Heart Center, Faculty of Medicine, University of Cologne, Cologne, Germany; b Cardiovascular Research Foundation, New York, New York, USA; c Center for Cardiovascular Medicine ABCD, Aachen – Bonn – Cologne-Düsseldorf, Germany; d Department of Cardiology and Vascular Medicine, West German Heart and Vascular Center, University Hospital Essen, University of Duisburg Essen, Essen, Germany; e Clinic for General and Interventional Cardiology/Angiology, Herz- und Diabeteszentrum NRW, Ruhr-Universität Bochum, Bad Oeynhausen, Germany; f Herz- und Diabeteszentrum Nordrhein-Westfalen, Med. Fakultät OWL (Universität Bielefeld), Bad Oeynhausen, Germany; g Department of Internal Medicine/Cardiology, Heart Center Leipzig at University of Leipzig and Leipzig Heart Science, Leipzig, Germany; h Department of Cardiology, Pulmonology and Vascular Medicine, University Hospital Düsseldorf, Medical Faculty of the Heinrich Heine University Düsseldorf, Düsseldorf, Germany; i Department of Cardiology and Angiology, Justus-Liebig-University Giessen, Giessen, Germany; j Department of Internal Medicine II, Ulm University Heart Center, Ulm, Germany; k Medizinische Klinik und Poliklinik I, Ludwig-Maximilians-Universität München, Munich, Germany; l Munich Heart Alliance, Partner Site German Center for Cardiovascular Disease (DZHK), Munich, Germany; m Department of Cardiology, University Heart & Vascular Center Hamburg, University Medical Center Hamburg-Eppendorf, Hamburg, Germany; n Medical Clinic I, Department of Cardiology, St. Johannes Hospital, Dortmund, Germany; o Department of Internal Medicine III, Division of Cardiology, University Hospital Heidelberg, Ruprecht-Karls University Heidelberg, Heidelberg, Germany; p Department of Internal Medicine II, Heart Center Bonn, University Hospital Bonn, Bonn, Germany; q Department of Cardiology, University Medical Center of the Johannes Gutenberg-University Mainz, Mainz, Germany

**Keywords:** 3-dimensional, mitral regurgitation, MR, M-TEER, MVOA, PASCAL

## Abstract

**Background:**

Mitral valve transcatheter edge-to-edge repair (M-TEER) is an established therapy for patients with mitral regurgitation (MR) at high surgical risk. By reducing the mitral valve orifice area (MVOA), M-TEER may increase the risk of iatrogenic mitral stenosis. Data for PASCAL M-TEER according to 3-dimensional (3D)-MVOA remain limited.

**Objectives:**

The objective of the study was to compare procedural, echocardiographic, and clinical outcomes after PASCAL M-TEER according to baseline 3D-MVOA.

**Methods:**

Patients with available baseline 3D-MVOA from the investigator-initiated multicenter REPAIR registry were stratified by 3D-MVOA (<4 vs ≥4 cm^2^). Outcomes included Mitral Valve Academic Research Consortium–defined technical success, MR ≤1+ at discharge, ≥1 NYHA functional class improvement at 30-days, and 1-year all-cause mortality.

**Results:**

Among 1,189 patients (mean age 78 ± 9 years, 44% female, 86% NYHA functional class III/IV, mean 3D-MVOA 4.9 ± 1.6 cm^2^, 45% secondary MR), 338 (28%) had baseline 3D-MVOA <4 cm^2^ and 851 (72%) ≥4 cm^2^. Technical success was achieved in 95.9% vs 98.2% (*P* = 0.028) and MR reduction to ≤1+ in 72% vs 72% (*P* > 0.999), respectively. At 30 days, NYHA functional class improved in 61% vs 64% (*P* = 0.532). One-year all-cause mortality was 13.0% (95% CI: 8.5% to 17.4%) vs 13.6% (95% CI: 10.9% to 16.3%; *P* = 0.760); adjusted HR (<vs ≥ 4 cm^2^): 1.05 (95% CI: 0.67-1.63; *P* = 0.842).

**Conclusions:**

Almost one-third of patients with available measurements had baseline 3D-MVOA <4 cm^2^. Technical success was modestly lower, whereas MR reduction and 1-year clinical outcomes did not differ significantly. These data are consistent with the feasibility of PASCAL M-TEER in carefully selected patients with small 3D-MVOA at experienced centers.

Mitral valve transcatheter edge-to-edge repair (M-TEER) is an established therapeutic option for patients with symptomatic severe mitral regurgitation (MR) who are considered at prohibitive surgical risk.^1^^,^^2^ By approximating the anterior and posterior mitral valve leaflets, M-TEER inherently reduces the mitral valve orifice area (MVOA), thereby introducing a risk of iatrogenic mitral stenosis, particularly in patients with small baseline MVOA.^3^ Consequently, both historical and contemporary pivotal M-TEER trials have consistently applied an MVOA threshold of <4 cm^2^ as an exclusion criterion.^4^, ^5^, ^6^

Notwithstanding these exclusion criteria, several studies have explored M-TEER in patient populations considered at an increased risk of iatrogenic mitral valve stenosis.^7^, ^8^, ^9^ However, these studies have been confined to the MitraClip system (Abbott Structural), small, highly selected or historic cohorts, reliance on pressure half-time-derived mitral valve area assessment, or the use of composite anatomical definitions, thereby limiting their clinical applicability.^7^, ^8^, ^9^

Data on M-TEER with the PASCAL system (Edwards Lifesciences) in small mitral valve anatomies are currently limited to a single case series.^10^ Particularly in this setting, owing to its distinctive design features, including advanced steering capabilities, independent leaflet clasping, flexible nitinol paddles, and a central spacer, the PASCAL system may represent a technically attractive approach.^10^^,^^11^ Whether similar procedural, echocardiographic, and clinical outcomes can be achieved with the PASCAL system in patients with 3-dimensional (3D) MVOA <4 cm^2^ vs ≥4 cm^2^ remains uncertain. To address this knowledge gap, we analyzed data from the REPAIR (REgistry of PAscal for mItral Regurgitation study DRKS00033959), a multicenter registry of patients undergoing PASCAL M-TEER.

## Methods

### Study design and patient population

The REPAIR registry's design and methodology have been previously published.^12^ In brief, REPAIR is an ongoing, investigator-initiated, multicenter observational registry including patients with MR undergoing M-TEER with the PASCAL system from 2019 through 2024 across 14 participating institutions. Patient selection was determined by multidisciplinary heart team evaluation at each site, with eligibility based on clinical presentation, anatomical feasibility, and optimization of guideline-directed medical therapy.

All procedures utilized the PASCAL system following previously established implantation protocols.^6^^,^^11^ The registry spans the full evolution of the device platform and includes patients treated when only the PASCAL P10 was available, when PASCAL P10 and Ace were available, and when the system was upgraded to the PASCAL Precision system with both devices available.^13^ Given the lack of standardized selection criteria, the decision to use PASCAL vs alternative M-TEER devices was made according to institutional preference and operator judgment. Echocardiographic evaluation was performed by experienced echocardiographers at participating centers following current guidelines. MR assessment was conducted at baseline, hospital discharge, 30-day follow-up, and subsequent visits using comprehensive multiparametric evaluation. MR was categorized as none/trace (0), mild (1+), mild-to-moderate (2+), moderate-to-severe (3+), or severe (4+).^14^^,^^15^ MR etiology was categorized as primary, secondary, or mixed.^14^ Tricuspid regurgitation severity was similarly assessed through multiparametric evaluation and classified as none/trace, mild, moderate, or severe.^14^^,^^15^

This study adhered to Declaration of Helsinki principles, obtained approval from local institutional ethics committees, and is registered with the German Clinical Trials Register (DRKS00033959).

### Study definitions

For this analysis, only patients with available 3D-MVOA assessments were included, as obtained from either 3D transesophageal echocardiography using multiplanar reconstruction or direct 3D measurement according to local practice. Patients were stratified according to 3D-MVOA <4.0 vs ≥4.0 cm^2^.

### Study outcomes

The primary endpoint was technical success as defined by the Mitral Valve Academic Research Consortium (MVARC). Additional outcomes were achievement of MR ≤ 1+ at discharge, improvement in NYHA functional class by at least 1 class at 30 days, 1-year all-cause mortality, a composite of 1-year all-cause mortality or first hospitalization for heart failure (HF), and the 1-year reintervention rate. The durability of the result was assessed in a paired analysis. Sensitivity analyses for the MVARC-defined technical success and achievement of MR ≤ 1+ at discharge were performed by stratifying the cohort according to MR etiology, PASCAL era, and by applying alternative 3D-MVOA thresholds (<3.5 vs ≥3.5 cm^2^ and <3.0 vs ≥3.0 cm^2^). An exploratory analysis compared PASCAL P10-only and PASCAL Ace-only device strategies in patients with 3D-MVOA <4 cm^2^ treated during the Ace era.

### Statistical analysis

Data were summarized as mean ± SD, median (Q1–Q3), or frequencies (n [%]). Between-group comparisons were performed using the chi-square test or Fisher exact for categorical variables and the Mann-Whitney U-test for continuous variables, as appropriate. Time-to-event outcomes were assessed using the Kaplan-Meier method and compared between groups with the log-rank test and estimates are reported with 95% CIs. Time-to-event outcomes were further analyzed using Cox proportional hazards regression models and were estimated as mixed-effects with random intercept for center in all analyses. Proportional hazards were verified using Schoenfeld residuals. Multivariable Cox regression models included age, sex, NYHA functional class, arterial hypertension, atrial fibrillation, coronary artery disease, diabetes mellitus, MR etiology, baseline MR grade, left ventricular ejection fraction, and dichotomized 3D-MVOA (<4 vs ≥4 cm^2^). Effect estimates are reported as HRs with 95% CIs. The binary endpoints of MVARC-defined technical success and MR reduction to ≤1+ at discharge were additionally analyzed using mixed-effects logistic regression with random intercept for center, with results reported as OR with 95% CIs. To address potential temporal confounding from device platform evolution, this model was additionally adjusted for PASCAL device era. Baseline characteristics between patients with and without available 3D-MVOA measurements were compared using standardized mean differences to assess covariate balance independent of sample size, with values <0.10 indicating negligible imbalance. Two-sided *P* values <0.05 were considered statistically significant and were not adjusted for multiplicity. Analyses were performed using complete cases, and missing data were not imputed. All statistical analyses were conducted using R (version 4.5.1; R Foundation for Statistical Computing).

## Results

### Baseline characteristics

Of the 2,601 patients included in the REPAIR registry, 1,189 (45.7%) had available baseline 3D-MVOA measurements and constituted the study population. The mean age was 78.0 ± 9.4 years, 44% were females, 86% were in NYHA functional class III or IV, and 45% had secondary MR. The median follow-up was 339 days (Q1-Q3: 70-540 days). Baseline characteristics are summarized in Table 1.

**Table 1:** Baseline Characteristics According to Baseline 3D-MVOA

	Overall (N = 1,189)	MVOA <4 cm^2^ (n = 338)	MVOA ≥4 cm^2^ (n = 851)	*P* Value
Age, y	80 [75-84] (n = 1,189)	81 [77-85] (n = 338)	80 [74-84] (n = 851)	**0.019**
Female	522/1,189 (43.9)	206/338 (60.9)	316/851 (37.1)	**<0.001**
Body mass index, kg/m^2^	25 [23-28.0] (n = 1,187)	25 [23-28] (n = 337)	25 [23-28] (n = 850)	0.063
NYHA functional class				0.972
I	10/1,189 (0.8)	3/338 (0.9)	7/851 (0.8)	
II	160/1,189 (13.5)	43/338 (12.7)	117/851 (13.7)	
III	881/1,189 (74.1)	252/338 (74.6)	629/851 (73.9)	
IV	138/1,189 (11.6)	40/338 (11.8)	98/851 (11.5)	
NT-proBNP, pg/mL	2,584 [1,199-5,366] (n = 924)	2,539 [1,118-5,244] (n = 251)	2,590 [1,240-5,411] (n = 673)	0.571
EuroSCORE II, %	4.5 [2.8-7.8] (n = 1,189)	4.9 [2.8-7.9] (n = 338)	4.4 [2.9-7.7] (n = 851)	0.506
Comorbidities				
Arterial hypertension	967/1,189 (81.3)	291/338 (86.1)	676/851 (79.4)	**0.010**
Diabetes mellitus	283/1,188 (23.8)	94/338 (27.8)	189/850 (22.2)	0.050
Coronary artery disease	678/1,189 (57.0)	209/338 (61.8)	469/851 (55.1)	**0.041**
Previous myocardial infarction	209/1,188 (17.6)	63/338 (18.6)	146/850 (17.2)	0.608
Previous cardiac surgery	219/1,189 (18.4)	53/338 (15.7)	166/851 (19.5)	0.146
Previous stroke/TIA	123/1,161 (10.6)	36/336 (10.7)	87/825 (10.5)	>0.999
CRT/ICD/PM	275/1,189 (23.1)	72/338 (21.3)	203/851 (23.9)	0.387
Atrial fibrillation	871/1,189 (73.3)	233/338 (68.9)	638/851 (75.0)	**0.041**
Chronic lung disease	247/1,189 (20.8)	69/338 (20.4)	178/851 (20.9)	0.910
Renal function				
eGFR, mL/min	48 [36-67] (n = 1,184)	46 [35-69] (n = 338)	49 [36-66] (n = 846)	0.758
eGFR <60 mL/min	800/1,184 (67.6)	220/338 (65.1)	580/846 (68.6)	0.279
On dialysis	26/1,086 (2.4)	7/281 (2.5)	19/805 (2.4)	>0.999
Medication				
ACEI, ARB, or ARNI	712/961 (74.1)	210/293 (71.7)	502/668 (75.1)	0.292
ARNI	194/781 (24.8)	49/231 (21.2)	145/550 (26.4)	0.153
Beta blocker	813/961 (84.6)	238/293 (81.2)	575/668 (86.1)	0.069
MRA	453/961 (47.1)	121/293 (41.3)	332/668 (49.7)	**0.020**
SGLT2 inhibitors	297/920 (32.3)	82/285 (28.8)	215/635 (33.9)	0.147
Loop diuretic	827/961 (86.1)	243/293 (82.9)	584/668 (87.4)	0.080

Values are n/N (%) or median [Q1-Q3] (n). **Bold** values indicate statistical significance.

3D = 3-dimensional; ACEI = angiotensin converting enzyme inhibitor; ARB = angiotensin receptor blocker; ARNI = angiotensin receptor neprilysin inhibitor; CRT = cardiac resynchronization therapy; eGFR = estimated glomerular filtration rate (calculated using the Cockcroft–Gault equation); EuroSCORE II = European System for Cardiac Operative Risk Evaluation II; ICD = implantable cardioverter-defibrillator; MRA = mineralocorticoid receptor antagonist; MVOA = mitral valve orifice area; NT-proBNP = N-terminal pro–B-type natriuretic peptide; PM = pacemaker; SGLT2 = sodium-glucose cotransporter-2; TIA = transient ischemic attack.

Among included patients, the mean 3D-MVOA was 4.9 ± 1.6 cm^2^. Of these, 338 (28.4%) had 3D-MVOA <4 cm^2^ and 851 (71.6%) had 3D-MVOA ≥4 cm^2^. Compared with patients with baseline 3D-MVOA ≥4 cm^2^, those with smaller 3D-MVOA were older, more frequently females, had a higher prevalence of arterial hypertension, diabetes, and coronary artery disease, whereas atrial fibrillation was more prevalent in patients with larger 3D-MVOA. Baseline N-terminal pro–B-type natriuretic peptide levels, surgical risk according to European System for Cardiac Operative Risk Evaluation II, renal function, and the proportion of patients in NYHA functional class ≥III were similar between groups.

Echocardiographic baseline characteristics are described in Table 2. Patients with baseline 3D-MVOA <4 cm^2^ had smaller left atrial and left ventricular dimensions and higher left ventricular ejection fraction. MR etiology differed between groups (*P* < 0.001), with secondary MR being less frequent in the <4 cm^2^ group (36% vs 49%; *P* < 0.001) and mixed MR being more prevalent (28% vs 17%; *P* < 0.001). The distribution of MR grade also differed between groups, with a higher proportion of MR grade 3+ in patients with baseline 3D-MVOA <4 cm^2^.

**Table 2:** Baseline Echocardiographic Characteristics According to Baseline 3D-MVOA

	Overall (N = 1,189)	MVOA <4 cm^2^ (n = 338)	MVOA ≥4 cm^2^ (n = 851)	*P* Value
Left heart				
LAVi, mL/m^2^	61 [48-80] (n = 993)	54 [43-71] (n = 272)	63 [50-84] (n = 721)	**<0.001**
LVEF, %	53 [39-59] (n = 1,136)	55 [42-60] (n = 308)	51 [37-58] (n = 828)	**0.013**
LVEDD, mm	54 [48-62] (n = 1,044)	51 [46-57] (n = 269)	56 [49-64] (n = 775)	**<0.001**
LVESD, mm	40 [33-50] (n = 865)	37 [30-45] (n = 198)	42 [34-52] (n = 667)	**<0.001**
LVESD <35 mm	271/865 (31.3)	79/198 (39.9)	192/667 (28.8)	**0.004**
LVEDVi, mL/m^2^	70 [54-92] (n = 860)	66 [51-83] (n = 211)	72 [55-95] (n = 649)	**0.003**
Mitral valve				
MR etiology				**<0.001**
PMR	415/1,188 (34.9)	122/337 (36.2)	293/851 (34.4)	0.610
SMR	534/1,188 (44.9)	120/337 (35.6)	414/851 (48.6)	**<0.001**
MMR	239/1,188 (20.1)	95/337 (28.2)	144/851 (16.9)	**<0.001**
MPG, mm Hg	2.0 [1.0-2.7] (n = 1,142)	2.0 [1.2-3.0] (n = 318)	2.0 [1.0-2.5] (n = 824)	**<0.001**
3D-MVOA, cm^2^	4.7 [3.8-5.8] (n = 1,189)	3.4 [3.0-3.7] (n = 338)	5.2 [4.5-6.3] (n = 851)	**<0.001**
VC width, mm	8.0 [6.0-9.0] (n = 1,060)	7.3 [6.0-9.0] (n = 282)	8.0 [6.3-9.0] (n = 778)	**0.018**
EROA, mm^2^	31 [23-45] (n = 1,112)	30 [20-42] (n = 313)	32 [25-47] (n = 799)	**<0.001**
Regurgitant volume, mL	46 [35-62] (n = 945)	45 [34-61] (n = 258)	47 [35-62] (n = 687)	0.390
MR grade				**0.036**
0+	0/1,189 (0.0)	0/338 (0.0)	0/851 (0.0)	
1+	0/1,189 (0.0)	0/338 (0.0)	0/851 (0.0)	
2+	23/1,189 (1.9)	3/338 (0.9)	20/851 (2.4)	0.108
3+	661/1,189 (55.6)	205/338 (60.7)	456/851 (53.6)	**0.032**
4+	505/1,189 (42.5)	130/338 (38.5)	375/851 (44.1)	0.089
Right heart				
TR grade				0.104
None/trace	30/1,077 (2.8)	6/279 (2.2)	24/798 (3.0)	0.591
Mild	368/1,077 (34.2)	85/279 (30.5)	283/798 (35.5)	0.149
Moderate	391/1,077 (36.3)	118/279 (42.3)	273/798 (34.2)	**0.019**
Severe	288/1,077 (26.7)	70/279 (25.1)	218/798 (27.3)	0.519
TAPSE, mm	18 [15-21] (n = 1,080)	18 [15-22] (n = 319)	18 [15-21] (n = 761)	0.946
PASP, mmHg	46 [36-57] (n = 1,125)	46 [37-58] (n = 318)	45 [36-56] (n = 807)	0.293

Values are n/N (%) or median [Q1-Q3] (n). **Bold** values indicate statistical significance.

EROA = effective regurgitant orifice area; LAVi = left atrial volume index; LVEF = left ventricular ejection fraction; LVEDD = left ventricular end diastolic diameter; LVEDVi = left ventricular end diastolic volume index; LVESD = left ventricular end systolic diameter; MMR = mixed mitral regurgitation; MPG = mean transmitral pressure gradient; MR = mitral regurgitation; PASP = pulmonary artery systolic pressure; PMR = primary MR; SMR = secondary MR; TAPSE = tricuspid annular plane systolic excursion; TR = tricuspid regurgitation; VC = vena contracta; other abbreviations as in Table 1.

The mean transmitral pressure gradients were higher in patients with smaller baseline 3D-MVOA (2.3 ± 1.2 mm Hg vs 2.0 ± 1.0 mm Hg; *P* < 0.001), without differences in gradients ≥5 mm Hg. Baseline 3D-MVOA differed substantially by group definition (3.3 ± 0.6 cm^2^ vs 5.6 ± 1.4 cm^2^; *P* < 0.001). Right-sided echocardiographic parameters were similar between groups.

Clinical and echocardiographic baseline characteristics of patients with and without available 3D-MVOA measurements are summarized in Supplemental Tables 1 and 2. Overall, baseline characteristics were well balanced between included and excluded patients, with the majority of standardized mean differences below 0.10. However, residual imbalances were observed for selected echocardiographic variables, including MR etiology, effective regurgitant orifice area, and regurgitant volume.

### Outcomes

#### Procedural outcomes

Procedural outcomes are summarized in Table 3. Technical success was achieved in 95.9% of patients with baseline 3D-MVOA <4 cm^2^ and in 98.2% of patients with 3D-MVOA ≥4 cm^2^ (*P* = 0.028) (Central Illustration). This lower rate in the <4 cm^2^ group was also observed in a mixed-effects logistic regression model with random intercept for center (adjusted OR: 0.33; 95% CI 0.15-0.71; *P* = 0.005) and was primarily driven by lower rates of successful deployment and correct positioning of the first intended device (96.7% vs 98.9%; *P* = 0.027). Other components of the MVARC-defined technical success endpoint were numerically similar between groups. Details based on the MVARC definition for technical success are provided in Supplemental Table 3 and narratives are provided in Supplemental Table 4.

**Table 3:** Procedural Outcomes According to Baseline 3D-MVOA

	Overall (N = 1,189)	MVOA <4 cm^2^ (n = 338)	MVOA ≥4 cm^2^ (n = 851)	*P* Value
Technical success	1,160/1,189 (97.6)	324/338 (95.9)	836/851 (98.2)	**0.028**
Intraprocedural device detachment	4/1,189 (0.3)	2/338 (0.6)	2/851 (0.2)	0.321
Partial	2/1,189 (0.2)	2/338 (0.6)	0/851 (0.0)	0.081
SLDA	2/1,189 (0.2)	0/338 (0.0)	2/851 (0.2)	>0.999
Procedure aborted	18/1,189 (1.5)	9/338 (2.7)	9/851 (1.1)	0.075
Leaflet damage	4/1,189 (0.3)	2/338 (0.6)	2/851 (0.2)	0.321
Devices implanted				**<0.001**
1	787/1,189 (66.2)	257/338 (76.0)	530/851 (62.3)	
2	363/1,189 (30.5)	70/338 (20.7)	293/851 (34.4)	
3	21/1,189 (1.8)	2/338 (0.6)	19/851 (2.2)	
4	0/1,189 (0.0)	0/338 (0.0)	0/851 (0.0)	
Number of devices implanted	1 [1-2] (n = 1,189)	1 [1-1] (n = 338)	1 [1-2] (n = 851)	**<0.001**
Device type				**<0.001**
P10 only	422/1,189 (35.5)	112/338 (33.1)	310/851 (36.4)	0.316
Ace only	711/1,189 (59.8)	215/338 (63.6)	496/851 (58.3)	0.104
Both P10 and Ace	38/1,189 (3.2)	2/338 (0.6)	36/851 (4.2)	**<0.001**
Device era				
P10	298/1,189 (25.1)	62/338 (18.3)	236/851 (27.7)	**0.001**
P10 and Ace	393/1,189 (33.1)	120/338 (35.5)	273/851 (32.1)	0.288
Precision	498/1,189 (41.9)	156/338 (46.2)	342/851 (40.2)	0.069
Independent clasping	389/771 (50.5)	87/201 (43.3)	302/570 (53.0)	**0.022**
Procedure duration, min	76 [56-104] (n = 1,110)	70 [55-96] (n = 315)	78 [58-106] (n = 795)	**0.005**
Postprocedural 3D-MVOA, cm^2^	2.3 (1.9-3.0) (n = 517)	1.7 (1.5-2.0 (n = 96)	2.5 (2.0-3.2 (n = 421)	**<0.001**

Values are n/N (%) or median [Q1-Q3] (n). **Bold** values indicate statistical significance.

SLDA = single leaflet device attachment; other abbreviations as in Table 1.

**Central Illustration:**
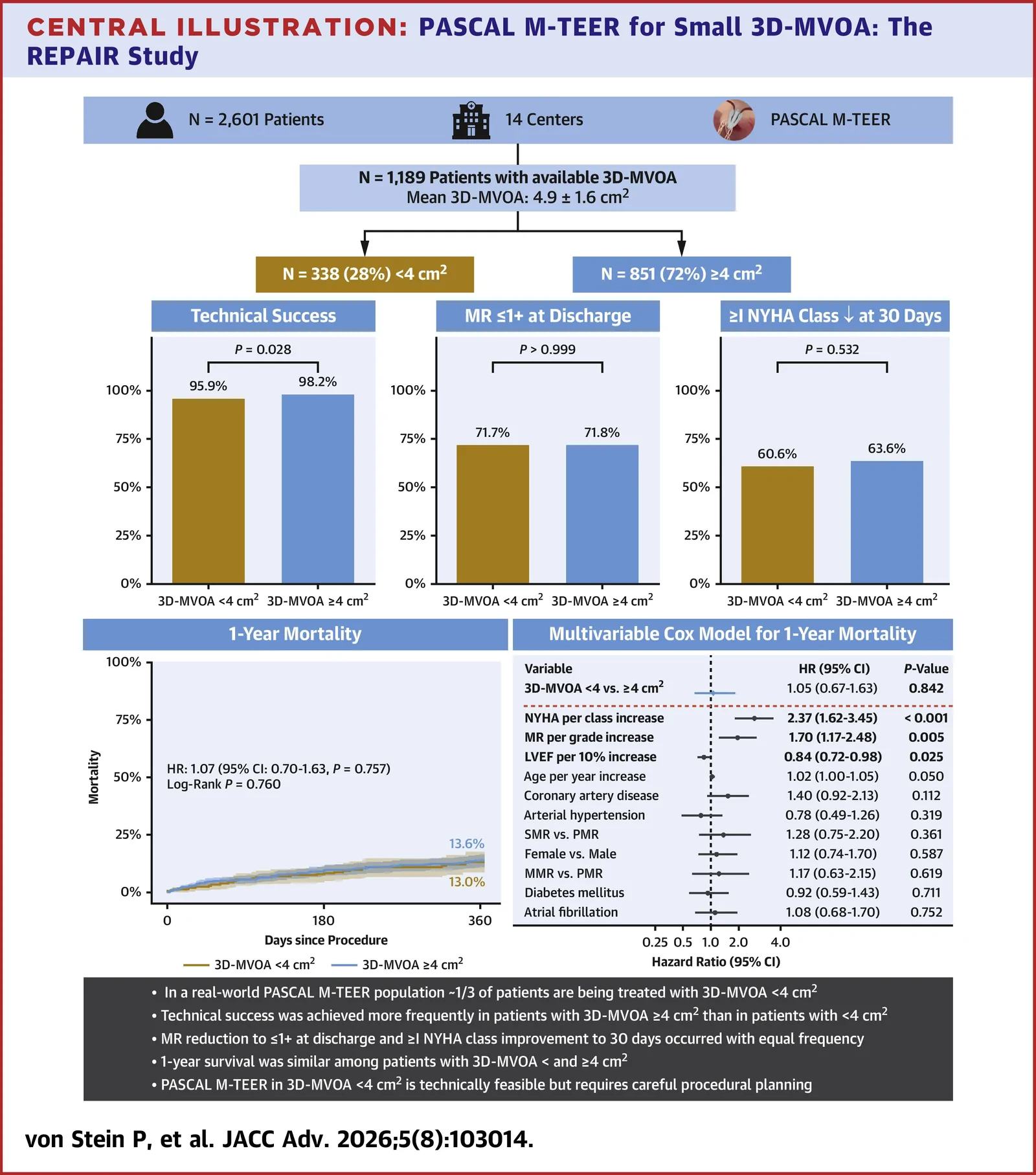
PASCAL M-TEER for Small 3D-MVOA: The REPAIR Study Patients undergoing M-TEER were stratified according to baseline 3D-MVOA. Of the 1,189 patients, 28% were treated with 3D-MVOA <4 cm^2^ and 72% with 3D-MVOA ≥4 cm^2^. MVARC-defined technical success (95.9% vs 98.2%; *P* = 0.028), MR reduction to MR ≤1+ (71.7% vs 71.8%; *P* > 0.999), NYHA functional class reduction by ≥I class to 30 days (60.6% vs 63.6%; *P* = 0.532), 1-year all-cause mortality (13.0% vs 13.6%; *P* = 0.760), and a multivariable Cox model for 1-year all-cause mortality (HR 1.05, 95% CI: 0.67-1.63, *P* = 0.842) are shown for patients with 3D-MVOA <4 compared to ≥4 cm^2^, respectively. 3D = 3-dimensional; LVEF = left ventricular ejection fraction; MMR = mixed MR; MR = mitral regurgitation; M-TEER = mitral valve transcatheter edge-to-edge repair; MVOA = mitral valve orifice area; PMR = primary MR; SMR = secondary MR.

Fewer devices were implanted in patients with baseline 3D-MVOA <4 cm^2^, with a higher proportion treated with a single device. Device selection did not differ between PASCAL P10 (33% vs 36%; *P* = 0.316) and PASCAL Ace (64% vs 58%; *P* = 0.104). However, in the subgroup of patients treated during the period when both PASCAL P10 and Ace were available (n = 891), exclusive use of the PASCAL P10 was more common among patients with 3D-MVOA <4 cm^2^ than among those with 3D-MVOA ≥4 cm^2^ (19% [53/276] vs 13% [79/615]; *P* = 0.018) (Supplemental Table 5). Independent clasping was more frequently used in patients with 3D-MVOA ≥4 cm^2^, and procedure duration was longer in this group. Postprocedural 3D-MVOA was available in 517 patients and was smaller in patients with baseline 3D-MVOA <4 cm^2^ than in those with ≥4 cm^2^ (1.7 [1.5-2.0] vs 2.5 [2.0-3.2] cm^2^; *P* < 0.001).

#### Echocardiographic outcomes

Echocardiographic outcomes are summarized in Table 4. At discharge, reduction of MR to ≤1+ was achieved in 72% of patients, with no statistically significant difference between patients with baseline 3D-MVOA <4 cm^2^ and ≥4 cm^2^ (71.7% vs 71.8%; *P* > 0.999) (Central Illustration). This finding was consistent in a mixed-effects logistic regression with random intercept for center (adjusted OR: 0.89; 95% CI 0.66-1.21; *P* = 0.462). The mean transmitral gradients at discharge were higher in patients with smaller baseline 3D-MVOA (3.8 ± 1.7 mmHg vs 3.3 ± 1.5 mmHg; *P* < 0.001), and gradients ≥5 mmHg occurred more frequently in this group (25% vs 16%; *P* < 0.001).

**Table 4:** Echocardiographic and Clinical Outcomes According to Baseline 3D-MVOA

	Overall (N = 1,189)	MVOA <4 cm^2^ (n = 338)	MVOA ≥4 cm^2^ (n = 851)	*P* Value
Echocardiographic outcomes				
Discharge				
MR ≤2+	1,118/1,161 (96.3)	313/325 (96.3)	805/836 (96.3)	>0.999
MR ≤1+	833/1,161 (71.7)	233/325 (71.7)	600/836 (71.8)	>0.999
MPG, mm Hg	3.0 [2.3-4.0] (n = 1,119)	3.0 [2.8-4.8] (n = 318)	3.0 [2.1-4.0] (n = 801)	**<0.001**
≥5 mm Hg	204/1,119 (18.2)	78/318 (24.5)	126/801 (15.7)	**<0.001**
30 days				
MR ≤2+	717/761 (94.2)	185/202 (91.6)	532/559 (95.2)	0.090
MR ≤1+	515/761 (67.7)	129/202 (63.9)	386/559 (69.1)	0.206
MPG, mm Hg	3.0 [2.1-4.0] (n = 740)	4.0 [3.0-5.0] (n = 194)	3.0 [2.0-4.0] (n = 546)	**<0.001**
≥5 mm Hg	123/740 (16.6)	51/194 (26.3)	72/546 (13.2)	**<0.001**
Latest follow-up				
MR ≤2+	504/536 (94.0)	135/140 (96.4)	369/396 (93.2)	0.236
MR ≤1+	343/536 (64.0)	87/140 (62.1)	256/396 (64.6)	0.669
MPG, mm Hg	3.0 [2.2-4.1] (n = 532)	3.8 [2.8-5.0] (n = 138)	3.0 [2.1-4.0] (n = 394)	**0.003**
≥5 mm Hg	103/532 (19.4)	37/138 (26.8)	66/394 (16.8)	**0.014**
Clinical outcomes				
NYHA functional class at 30 days				0.861
I	122/734 (16.6)	30/188 (16.0)	92/546 (16.8)	
II	366/734 (49.9)	92/188 (48.9)	274/546 (50.2)	
III	230/734 (31.3)	63/188 (33.5)	167/546 (30.6)	
IV	16/734 (2.2)	3/188 (1.6)	13/546 (2.4)	
NYHA functional class at latest follow-up				0.114
I	99/593 (16.7)	15/142 (10.6)	84/451 (18.6)	
II	273/593 (46.0)	73/142 (51.4)	200/451 (44.3)	
III	207/593 (34.9)	50/142 (35.2)	157/451 (34.8)	
IV	14/593 (2.4)	4/142 (2.8)	10/451 (2.2)	

Values are n/N (%) or median (Q1-Q3) (n). **Bold** values indicate statistical significance.

Abbreviations as in Table 1.

**Figure 1:**
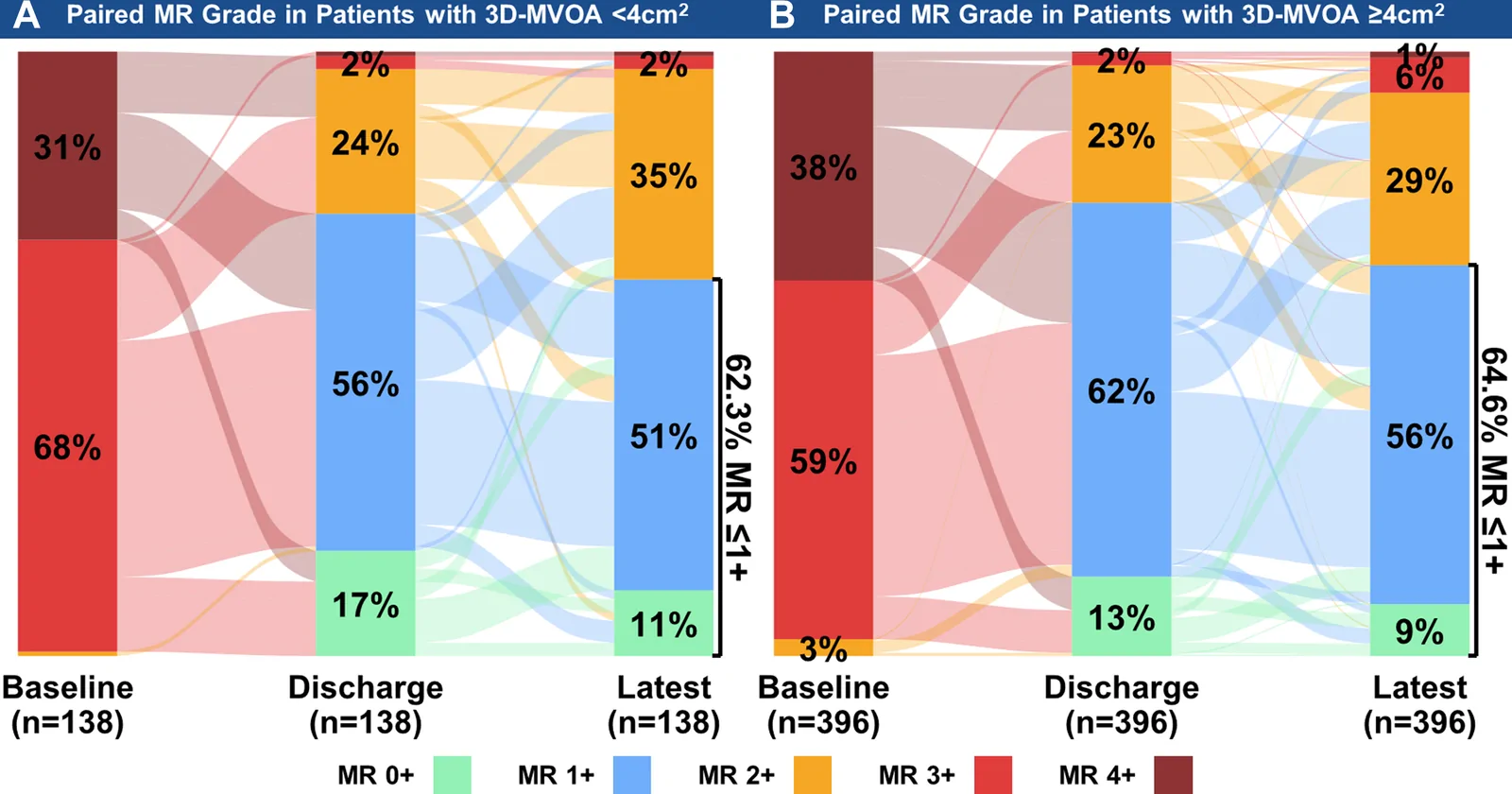
Paired MR Grade from Baseline to Latest Follow-Up Paired MR grade is depicted for (A) patients with 3D-MVOA <4 cm^2^ and (B) ≥4 cm^2^ with available echocardiographic assessments at baseline, discharge, and latest follow-up. Values < 1% are not displayed in the figure. In patients with 3D-MVOA <4 cm^2^ (A), 1 patient (0.7%, 1/138) had MR 2+ at baseline, 1 patient (0.7%, 1/138) had MR 4+ at discharge, and one patient (0.7%, 1/138) had MR 4+ at latest available echocardiographic follow-up. In patients with 3D-MVOA ≥4 cm^2^ (B), 1 patient (0.3%, 1/396) had MR 4+ at discharge. No difference was observed in the rate of MR ≤ 1+ at latest available echocardiographic follow-up (*P* = 0.698). 3D = three-dimensional; MR = mitral regurgitation; MVOA = mitral valve orifice area.

These findings remained consistent at 30-day echocardiographic follow-up, available in 64% of patients (766/1,189) after a median of 49 days (Q1-Q3: 33-92 days), and at the longest available echocardiographic follow-up, available in 45% of patients (536/1,189) after a median of 370 days (Q1-Q3: 342-573 days). Results were confirmed in paired analyses (Figure 1).

**Figure 4:**
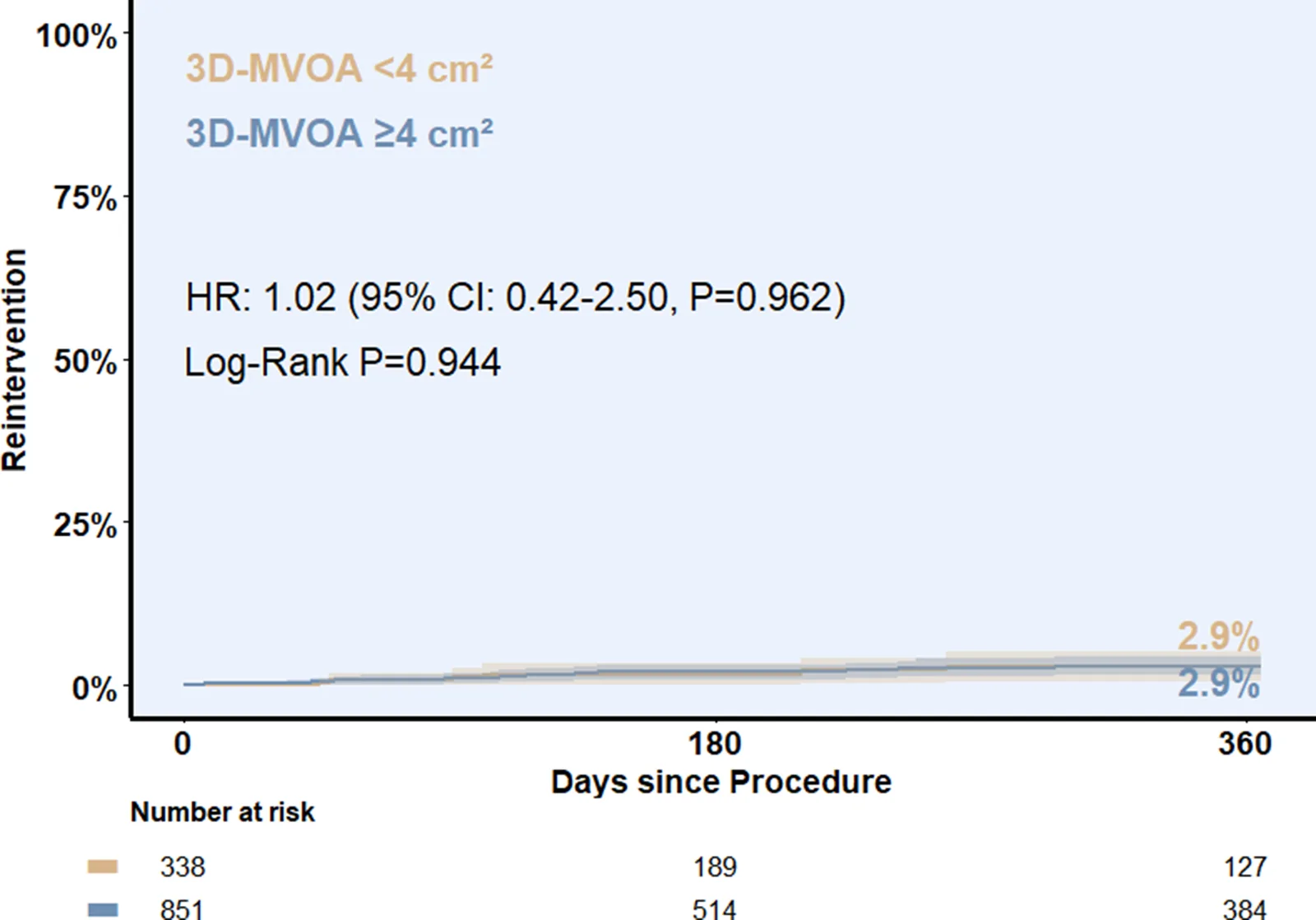
1-Year Mitral Valve Reintervention Rate According to Baseline 3D-MVOA Kaplan-Meier curve illustrating the 1-year reintervention rate according to baseline 3D-MVOA. The 1-year mitral valve reintervention rate was 2.9% (95% CI: 0.5%-5.2%) in patients with baseline 3D-MVOA <4 cm^2^ and 2.9% (95% CI: 1.5%-4.3%) in patients with 3D-MVOA ≥4 cm^2^. Abbreviations as in Figure 1.

#### Clinical outcomes

Clinical outcomes are provided in Table 4. NYHA functional class at 30-day follow-up was available for 62% of patients (734/1,189) and improved by at least 1 class in 61% of patients with 3D-MVOA <4 cm^2^ compared to 64% of patients with 3D-MVOA ≥4 cm^2^ (*P* > 0.999) (Central Illustration). NYHA functional class ≤II was achieved in 65% vs 67% at 30 days (*P* = 0.655) and in 62% vs 63% at the longest available follow-up (*P* = 0.908).

**Figure 2:**
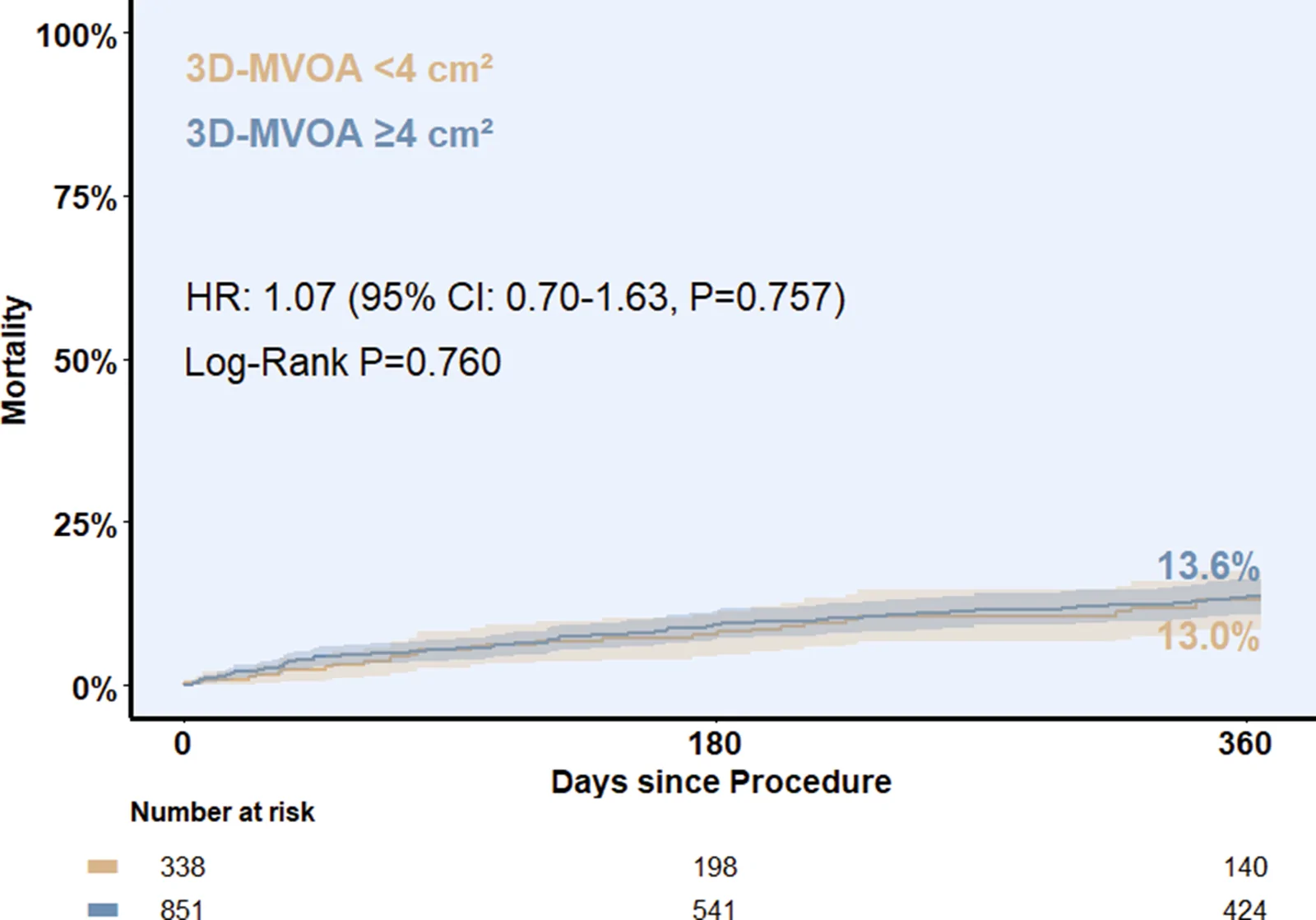
1-Year All-Cause Mortality According to Baseline 3D-MVOA Kaplan-Meier curve showing 1-year all-cause mortality according to baseline 3D-MVOA. One-year all-cause mortality was 13.0% (95% CI: 8.5%-17.4%) in patients with 3D-MVOA <4 cm^2^ and 13.6% (95% CI: 10.9%-16.3%) in those with 3D-MVOA ≥4 cm^2^ (*P* = 0.760). 3D = three-dimensional; MVOA = mitral valve orifice area.

**Figure 3:**
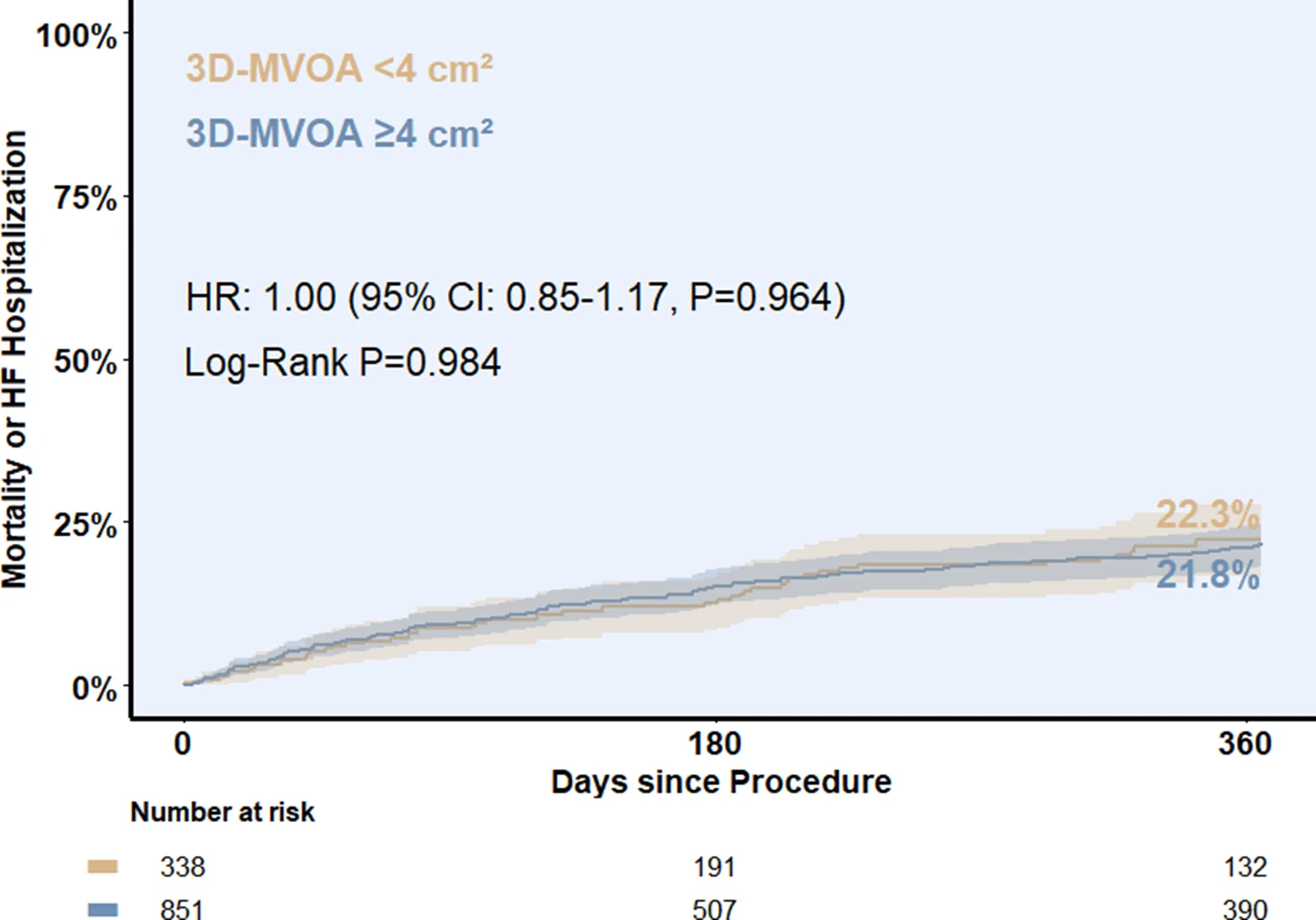
1-Year All-Cause Mortality or First Heart Failure Hospitalization According to Baseline 3D-MVOA Kaplan-Meier curve depicting 1-year all-cause mortality or heart failure hospitalization according to baseline 3D-MVOA. One-year all-cause mortality or first hospitalization for HF rates were 22.3% (95% CI: 16.6%-27.6%) in patients with baseline 3D-MVOA <4 cm^2^ and 21.8% (95% CI: 18.4%-25.0%) in those with 3D-MVOA ≥4 cm^2^ (*P* = 0.984). HF = heart failure; other abbreviations as in Figure 1.

Within 1-year, 117 patients died and 191 patients either died or were hospitalized for HF. No statistically significant between-group differences were observed in 1-year all-cause mortality, including after multivariable adjustment (Figure 2, Central Illustration). Similarly, no statistically significant between-group differences were observed for the composite of 1-year all-cause mortality or first hospitalization for HF (Figure 3), or the 1-year reintervention rate (Figure 4).

### Sensitivity analyses

Sensitivity analyses across MR etiologies were directionally consistent with the main analysis (Supplemental Table 6). When applying alternative 3D-MVOA thresholds, 184 of 1,189 patients (15.5%) had a baseline 3D-MVOA <3.5 cm^2^ and 78 (6.6%) had a baseline 3D-MVOA <3.0 cm^2^. Technical success was 97.3% vs 97.6% for patients with 3D-MVOA <3.5 vs ≥3.5 cm^2^ (*P* = 0.995) and 97.4% vs 97.6% for patients with 3D-MVOA <3.0 vs ≥3.0 cm^2^ (*P* = 0.715), with no statistically significant differences in MR reduction to ≤1+ at discharge across both thresholds (Supplemental Table 7). After additionally adjusting for PASCAL device era, results were directionally unchanged for both technical success and MR reduction to ≤1+ at discharge (Supplemental Table 8).

### Comparing PASCAL P10 and Ace in patients with 3D-MVOA <4 cm^2^

In an exploratory analysis of patients with 3D-MVOA <4 cm^2^ treated in the Ace era, PASCAL P10-only (n = 53) and PASCAL Ace-only (n = 215) strategies were compared. MVARC-defined technical success was lower with PASCAL P10 than PASCAL Ace (94.3% vs 99.5%; *P* = 0.025), reflecting 3 vs 1 patients not meeting MVARC-defined technical success criteria. No statistically significant differences were observed in MR reduction to ≤1+ at discharge (71.7% vs 73.0%; *P* = 0.987) or mean transmitral gradients at discharge (3.2 [2.8-4.2] vs 3.0 [3.0-4.9] mmHg; *P* = 0.890).

## Discussion

To the best of our knowledge, this analysis from the REPAIR study represents the largest comparison of patients undergoing PASCAL M-TEER according to baseline 3D-MVOA. This analysis has several key findings. First, nearly one-third of patients with available 3D-MVOA measurements were treated despite baseline 3D-MVOA <4 cm^2^. Second, although overall MVARC-defined technical success was high, it was less frequently achieved in patients with 3D-MVOA <4 cm^2^, primarily driven by successful deployment and correct positioning of the first intended device. This difference was not observed in sensitivity analyses using alternative MVOA thresholds of <3.5 cm^2^ and <3.0 cm^2^. Third, reduction to MR ≤1+ was achieved in 72% of patients, with no statistically significant difference according to baseline 3D-MVOA category. Fourth, no statistically significant differences in symptomatic improvement or 1-year clinical outcomes were observed between patients with 3D-MVOA <4 cm^2^ and those with ≥4 cm^2^.

The observation that nearly one-third of patients treated with PASCAL M-TEER had a baseline 3D-MVOA <4 cm^2^ is noteworthy and likely reflects contemporary real-world decision-making in experienced M-TEER centers. Importantly, this proportion probably underestimates the true prevalence of patients with small MVOA among patients evaluated for transcatheter therapy, as REPAIR includes only those deemed suitable for an M-TEER attempt.

These findings align with recently published single-center data, where 22% of MitraClip-treated patients had 3D-MVOA <4 cm^2^.^9^ Similarly, the multicenter Japanese Optimized Catheter Valvular Intervention-Mitral (OCEAN-Mitral) registry reported 20% of patients with MVOA <4 cm^2^.^7^ In contrast, the EXPAND G4 registry reported fewer than 10% of evaluable patients as being at "risk of stenosis".^8^ However, EXPAND G4 included only patients scheduled to receive the MitraClip according to approved indications for use, which specify MVOA ≥4 cm^2^ as an anatomical consideration for optimal results, thereby potentially underestimating the incidence of small 3D-MVOA.^8^

Differences in MR etiology across studies further contextualize these findings, as etiological distribution differs substantially between cohorts. The study by Kharsa et al.^9^ reported primary MR in 54%, secondary MR in 36%, and mixed MR in 10% of cases, whereas OCEAN-Mitral documented 24% primary MR, 71% secondary MR, and 5% mixed MR.^7^ EXPAND G4 included 37% primary MR and 63% secondary MR, with mixed MR included in the primary MR population.^8^ The present study demonstrated 35% primary MR, 45% secondary MR, and 20% mixed MR, representing the highest proportion of mixed MR among available studies.

Mechanistically, determinants of reduced MVOA may differ between patients with primary or mixed MR and those with secondary MR. Despite both leaflets having similar area, the annulus-to-tip length of the anterior mitral leaflet exceeds that of the posterior leaflet, which is why restriction of anterior mitral leaflet motion may have a greater impact on MVOA than the more commonly observed posterior leaflet restriction.^16^ In primary and mixed MR, mitral annular and leaflet calcification may directly relate to annular narrowing from calcific burden, whereas extension of calcification into the anterior mitral leaflet may restrict mobility, thereby limiting MVOA.^16^^,^^17^ The association of mitral annular calcification with small MVOA aligns with the observed demographics of older, more frequently female patients with higher rates of arterial hypertension, more frequent coronary artery disease, and a trend toward higher diabetes mellitus prevalence in patients with 3D-MVOA <4 cm^2^ in our study.^17^^,^^18^ In secondary MR, subvalvular tethering and restricted anterior leaflet mobility may constrain mitral valve opening despite absence of intrinsic leaflet disease.

The relatively high proportion of patients with 3D-MVOA <4 cm^2^ emphasizes a substantial need for treatment options for patients not considered optimal for M-TEER. Emerging transcatheter mitral valve replacement (TMVR) options target patients deemed unsuitable for M-TEER. However, the first U.S. Food and Drug Administration–approved transseptal TMVR device, the SAPIEN M3 (Edwards Lifesciences), would have excluded 40% of patients with 3D-MVOA <4 cm^2^ based solely on left ventricular end-systolic dimension <35 mm, rendering them at increased risk for difficult encircling or implantation.^19^

In addition, patients with 3D-MVOA <4 cm^2^ would not have been included in the EVEREST II (Endovascular Valve Edge-to-Edge Repair Study) trial, the COAPT (Cardiovascular Outcomes Assessment of the MitraClip Percutaneous Therapy for Heart Failure Patients With Functional Mitral Regurgitation) trial, or the CLASP trial.^4^, ^5^, ^6^ Nevertheless, M-TEER can be performed with high rates of MVARC-defined technical success in patients with 3D-MVOA <4 cm^2^, albeit slightly lower than in patients with 3D-MVOA ≥4 cm^2^. Differences were driven by successful deployment and correct positioning of the first intended device, which is required to meet the MVARC definition for technical success.^20^ Other components of MVARC-defined technical success were numerically similar between groups. Therefore, these differences may also reflect uncertainty in device selection rather than an inability to achieve adequate MR reduction. In fact, in patients with 3D-MVOA <4 cm^2^, more devices were trialed, with procedures sometimes initiated with PASCAL P10 and sometimes with PASCAL Ace. Such uncertainties might explain why sensitivity analyses failed to confirm the main result. Indeed, 1 case series on PASCAL treatment in small MVOA including three patients reported device switching from PASCAL P10 to PASCAL Ace in 1 patient, therefore formally not fulfilling MVARC-defined technical success.^10^ Consistent with this observation, an exploratory analysis comparing PASCAL P10-only and PASCAL Ace-only strategies in patients with 3D-MVOA <4 cm^2^ treated in the Ace era demonstrated lower MVARC-defined technical success with PASCAL P10 than PASCAL Ace, whereas no statistically significant differences were observed in MR reduction to ≤1+ at discharge or mean transmitral gradients, although these analyses are limited by sample size.

Technical considerations in patients with small MVOA may favor the PASCAL P10, as its 5 mm spacer creates separation while allowing residual leaflet mobility, and its 10 mm paddles facilitate effective MR reduction.^10^^,^^21^ Conversely, the smaller PASCAL Ace may also be advantageous in confined MVOA with limited maneuvering space, occupying less space with its 2 mm spacer and 6 mm paddles, potentially allowing placement of two devices even in smaller anatomies. In addition, device elongation might help prevent entanglement in confined areas. Despite these considerations, data remain insufficient.

Whether device selection certainty differs for small 3D-MVOA with MitraClip remains to be determined, as previous MitraClip studies have not reported MVARC-defined technical success, instead relying on definitions including survival and MR reduction to ≤2+. In the current analysis, MR reduction to ≤1+ was achieved in approximately 72% of patients with no statistically significant difference between groups. These results parallel previously reported MitraClip discharge outcomes in patients with 3D-MVOA <4 cm^2^.^7^^,^^9^ Although these analyses focused on different M-TEER systems, the present findings are consistent with the feasibility of M-TEER in carefully selected patients with small MVOA.^7^, ^8^, ^9^ This is cautiously supported by observation of stable MR results in both 3D-MVOA <4 cm^2^ and ≥4 cm^2^ groups, where durability concerns might arise due to fewer implants used.^22^

Regarding symptomatic improvement and clinical outcomes, no statistically significant differences were observed in NYHA functional class improvement, 1-year all-cause mortality, or the composite of 1-year all-cause mortality or hospitalization for HF. These findings should not be interpreted as evidence of equivalence, but they are reassuring in the context of the limited applicability of other treatments, including TMVR, in this anatomical subset.

Important considerations when evaluating patients for M-TEER extend beyond 3D-MVOA. Heart team evaluation should carefully weigh all options including M-TEER, mitral valve surgical repair or replacement, TMVR, and conservative management. Although 3D-MVOA evaluation represents one cornerstone of assessment, small 3D-MVOA, despite association with higher postprocedural gradients, should not, in isolation, preclude M-TEER in otherwise suitable candidates.^9^

### Study Limitations

Although REPAIR represents the largest industry-independent study of patients undergoing PASCAL M-TEER, several limitations warrant consideration. First, as a registry, clinical data were obtained from local sites without independent adjudication, and echocardiographic assessments were performed by individual centers without core laboratory review. Second, 3D-MVOA was assessed either by multiplanar reconstruction or direct 3D planimetry according to local practice. Although these approaches demonstrate excellent agreement, minor systematic differences between techniques cannot be excluded.^23^ However, for MR reduction to ≤1+, the consistency of findings across alternative MVOA thresholds and MR etiology subgroups, together with the attenuation of the technical success difference at stricter thresholds, provides some reassurance against systematic misclassification bias around the 4.0 cm^2^ cutoff. Third, although this real-world registry includes M-TEER-treated patients, true prevalence of patients with 3D-MVOA <4 cm^2^ cannot be determined, as only patients deemed suitable for M-TEER were included, without considering unsuitable patients. Patient selection for 3D-MVOA measurement may have been influenced by etiology and MR severity, and those with visually adequate MVOA may not have received measurement, whereas those with uncertain assessment were more likely to undergo measurement. This is supported by standardized mean differences >0.1 for selected echocardiographic variables, including MR etiology, effective regurgitant orifice area, and regurgitant volume, and may have introduced selection bias. Residual selection bias due to unmeasured factors, including anatomical features influencing the decision to perform 3D-MVOA assessment, cannot be excluded. In addition, specific etiological factors such as calcification were unavailable, limiting interpretability. Fourth, follow-up was limited by large referral areas, with many patients receiving subsequent care from community cardiologists, creating potential selection bias by capturing data primarily from notably improved or significantly deteriorating cases. Fifth, comparisons that did not reach statistical significance should be interpreted as an absence of a detected difference rather than evidence of equivalence. The CIs around clinical outcomes do not exclude clinically relevant differences. Finally, the study was conducted exclusively in Germany, which may limit generalizability to regions with less M-TEER experience.

## Conclusions

In this large real-world analysis, nearly one-third of patients undergoing PASCAL M-TEER with available baseline 3D-MVOA measurement had a baseline 3D-MVOA <4 cm^2^. The modest reduction in MVARC-defined technical success was confined to successful deployment and correct positioning of the first intended device. No statistically significant differences were observed in MR reduction, symptomatic improvement, or 1-year clinical outcomes. These findings are consistent with the feasibility of PASCAL M-TEER in carefully selected patients with small baseline 3D-MVOA when performed at experienced centers.

Perspectives **COMPETENCY IN MEDICAL KNOWLEDGE:** In carefully selected patients with symptomatic MR and a small baseline 3D-MVOA (<4.0 cm²), PASCAL M-TEER was associated with high rates of technical success and MR reduction to ≤1+. Compared with patients with a baseline 3D-MVOA ≥4.0 cm², no statistically significant differences were observed in symptomatic improvement or 1-year clinical outcomes.

**TRANSLATIONAL OUTLOOK:** As mitral valve repair and replacement options continue to expand, refined patient selection tailored to the respective anatomy is of increasing importance. In this context, small baseline 3D-MVOA should be considered but should not, in isolation, deem patients unsuitable for M-TEER. Longer-term follow-up is needed to assess the durability of clinical and echocardiographic outcomes after M-TEER in patients with a small baseline 3D-MVOA.

## Funding support and author disclosures

Dr Stolz has received speaker honoraria from 10.13039/100006520Edwards Lifesciences. Dr Mahabadi has received honoraria, lecture fees, and/or grant support from 10.13039/100000042Amgen, 10.13039/501100022274Daiichi Sankyo, 10.13039/100006520Edwards Lifesciences, 10.13039/100004336Novartis, and 10.13039/100004339Sanofi, all unrelated to this work; and is co-founder of Mycor GmbH, a company focusing on AI-based EKG-algorithms. Dr Jennifer von Stein has received speaker honoraria from 10.13039/100006520Edwards Lifesciences. Dr Gerçek has served as consultant for 10.13039/100006520Edwards Lifesciences; and has received a research grant from the Ruhr-University Bochum as Advanced Clinician Scientist. Dr Rassaf has received honoraria, lecture fees, and grant support from 10.13039/100006520Edwards Lifesciences, 10.13039/100004325AstraZeneca, 10.13039/100004326Bayer, 10.13039/100004336Novartis, 10.13039/501100022535Berlin Chemie, Daiicho-Sankyo, 10.13039/100001003Boehringer Ingelheim, 10.13039/501100004191Novo Nordisk, Cardiac Dimensions, and 10.13039/100004319Pfizer, all unrelated to this work; and is co-founder of Bimyo GmbH, a company that develops cardioprotective peptides, co-founder of Mycor GmbH, a company focusing on AI-based EKG-algorithms, and co-founder of Yes2NO, developing nitric oxide-based treatments. Dr Lurz has received institutional fees and research grants from Abbott Cardiovascular, 10.13039/100006520Edwards Lifesciences, and 10.13039/100004374Medtronic. Dr Horn has received an unrestricted research grant from 10.13039/100006520Edwards Lifesciences; and speaking honoraria from Abbott Cardiovascular. Florian Schindhelm received speaker honoraria and travel expenses from 10.13039/100006520Edwards Lifesciences, MedMile, and Björn Steiger Stiftung. Dr Möllmann has received speaker honoraria from Abbott Cardiovascular and 10.13039/100006520Edwards Lifesciences. Dr Baldus has received honorarium for consultation by 10.13039/100006520Edwards Lifesciences; and was supported by the Deutsche Forschungsgemeinschaft, Bonn, Germany (397484323). Dr Volker Rudolph has received research grants and honoraria for consultation from 10.13039/100006520Edwards Lifesciences. Dr Hausleiter has received speaker honoraria from and serves as consultant 10.13039/100006520Edwards Lifesciences and TriCares. Dr Pfister has received honorarium for consultation by 10.13039/100006520Edwards Lifesciences. Dr Mauri has received speaker honoraria and travel compensation by 10.13039/100006520Edwards Lifesciences and was supported by the Deutsche Forschungsgemeinschaft, Bonn, Germany (397484323). All other authors have reported that they have no relationships relevant to the contents of this paper to disclose.

## Supplementary material

Supplemental Material

Pubmed Investigator List
