# Supplementary material for: Small Mitral Valve Orifice Area in Mitral Valve Transcatheter Edge-to-Edge Repair: The REPAIR Study
Source: JACC Adv. 2026 Jun 25;5(8):103014. doi: 10.1016/j.jacadv.2026.103014 (PMC13531619; doi:10.1016/j.jacadv.2026.103014)
Supplement: Supplementary file 1 [file mmc1.pdf]

Supplemental Material

Supplemental Table 1. Baseline Characteristics of Included and Excluded Patients

	Overall (N=2,601)	Included (N=1,189)	Excluded (N=1,412)	SMD
Age, years	80 [74-84] (n=2,601)	80 [75-84] (n=1,189)	80 [74-84] (n=1,412)	0.019
Sex, female	1,150/2,601 (44.2)	522/1189 (43.9)	628/1412 (44.5)	0.012
Body mass index, kg/m^2	25 [23-28] (n=2,598)	25 [23-28] (n=1,187)	25 [23-29] (n=1,411)	0.069
NYHA class				
I	19/2,598 (0.7)	10/1189 (0.8)	9/1,409 (0.6)	0.024
II	374/2,598 (14.4)	160/1189 (13.5)	214/1,409 (15.2)	0.049
III	1,848/2,598 (71.1)	881/1189 (74.1)	967/1,409 (68.6)	**0.121**
IV	357/2,598 (13.7)	138/1189 (11.6)	219/1,409 (15.5)	**0.114**
NT-proBNP, pg/mL	2,590 [1,233-5,688] (n=1,925)	2,584 [1,199-5,366] (n=924)	2,593 [1,270-6,041] (n=1,001)	0.036
EuroSCORE II, %	4.7 [2.9-7.9] (n=2,601)	4.5 [2.8-7.8] (n=1,189)	4.9 [2.9-8.0] (n=1,412)	0.030
**Comorbidities**				
Arterial hypertension	2163/2,592 (83.4)	967/1,189 (81.3)	1,196/1,403 (85.2)	**0.105**
Diabetes mellitus	604/2,600 (23.2)	283/1,188 (23.8)	321/1,412 (22.7)	0.026
Coronary artery disease	1539/2,601 (59.2)	678/1,189 (57.0)	861/1,412 (61.0)	0.080
Previous myocardial infarction	454/2,600 (17.5)	209/1,188 (17.6)	245/1,412 (17.4)	0.006
Previous cardiac surgery	492/2,601 (18.9)	219/1,189 (18.4)	273/1,412 (19.3)	0.023
Previous stroke/TIA	290/2,472 (11.7)	123/1,161 (10.6)	167/1,311 (12.7)	0.067
CRT/ICD/PM	596/2,601 (22.9)	275/1,189 (23.1)	321/1,412 (22.7)	0.009
Atrial fibrillation	1,854/2,601 (71.3)	871/1,189 (73.3)	983/1,412 (69.6)	0.080
Chronic lung disease	627/2,601 (24.1)	247/1,189 (20.8)	380/1,412 (26.9)	**0.144**
**Renal function**				
eGFR, mL/min	48 [35-65] (n=2,590)	48 [36-67] (n=1,184)	47 [35-63] (n=1,406)	**0.139**
eGFR <60 mL/min	1,803/2,590 (69.6)	800/1,184 (67.6)	1,003/1,406 (71.3)	0.082
On dialysis	55/2,397 (2.3)	26/1,086 (2.4)	29/1,311 (2.2)	0.012
**Medication**				
ACEi, ARB, or ARNI	1,654/2,289 (72.3)	712/961 (74.1)	942/1,328 (70.9)	0.070
ARNI	398/1,774 (22.4)	194/781 (24.8)	204/993 (20.5)	**0.103**
Beta blocker	1,918/2,289 (83.8)	813/961 (84.6)	1,105/1,328 (83.2)	0.038
MRA	997/2,289 (43.6)	453/961 (47.1)	544/1,328 (41.0)	**0.125**
SGLT2 inhibitors	558/2,241 (24.9)	297/920 (32.3)	261/1,321 (19.8)	**0.290**
Loop diuretic	1,933/2,289 (84.4)	827/961 (86.1)	1,106/1,328 (83.3)	0.077

Values are n/N (%) or median [Q1-Q3] (n). **Bold** values indicate SMD >0.1. *ACEi=angiotensin converting enzyme inhibitor; ARB=angiotensin receptor blocker; ARNI=angiotensin receptor neprilysin inhibitor; CRT=cardiac resynchronization therapy; eGFR=estimated glomerular filtration rate (calculated using the Cockcroft–Gault equation); EuroSCORE II=European System for Cardiac Operative Risk Evaluation II; ICD=implantable cardioverter-defibrillator; MRA=mineralocorticoid receptor antagonist; NT-proBNP=N-terminal pro–B-type natriuretic peptide; NYHA=New York Heart Association; PM=pacemaker; SGLT2=sodium-glucose cotransporter-2; SMD=standardized mean difference; TIA=transient ischemic attack*

Supplemental Table 2. Echocardiographic Baseline Characteristics of Included and Excluded Patients

	Overall (N=2,601)	Included (N=1,189)	Excluded (N=1,412)	SMD
**Left heart**				
LAVi, mL/m^2	58 [46-78] (n=2,019)	61 [48-80] (n=993)	56 [44-75] (n=1,026)	**0.122**
LVEF, %	50 [37-58] (n=2,391)	53 [39-59] (n=1,136)	50 [35-57] (n=1,255)	**0.132**
LVEDD, mm	55 [48-62] (n=2,057)	54 [48-62] (n=1,044)	55 [49-62] (n=1,013)	0.048
LVESD, mm	40 [33-51] (n=1,584)	40 [33-50] (n=865)	41 [34-51] (n=719)	0.057
LVEDVi, mL/m^2	68 [51-91] (n=1,707)	70 [54-92] (n=860)	66 [48-90] (n=847)	0.098
**Mitral valve**				
MR etiology				
PMR	848/2580 (32.9)	415/1188 (34.9)	433/1392 (31.1)	0.081
SMR	1307/2580 (50.7)	534/1188 (44.9)	773/1392 (55.5)	**0.212**
MMR	425/2580 (16.5)	239/1188 (20.1)	186/1392 (13.4)	**0.182**
MPG, mmHg	2.0 [1.0-2.8] (n=2,102)	2.0 [1.0-2.7] (n=1,142)	2.0 [1.0-3.0] (n=960)	0.004
VC width, mm	7.3 [6.0-9.0] (n=1,731)	8.0 [6.0-9.0] (n=1,060)	7.0 [6.0-8.0] (n=671)	**0.152**
EROA, mm^2	35 [25-48] (n=2,134)	31 [23-45] (n=1,112)	38 [27-50] (n=1,022)	**0.227**
Regurgitant volume, mL	50 [36-66] (n=1,826)	46 [35-62] (n=945)	55 [40-70] (n=881)	**0.294**
MR grade				
0+	0/2,597 (0.0)	0/1,189 (0.0)	0/1,408 (0.0)	
1+	0/2,597 (0.0)	0/1,189 (0.0)	0/1,408 (0.0)	
2+	40/2,597 (1.5)	23/1,189 (1.9)	17/1,408 (1.2)	0.059
3+	1,482/2,597 (57.1)	661/1,189 (55.6)	821/1,408 (58.3)	0.055
4+	1,075/2,597 (41.4)	505/1,189 (42.5)	570/1,408 (40.5)	0.040
**Right heart**				
TR grade				
None/trace	73/2,298 (3.2)	30/1,077 (2.8)	43/1,221 (3.5)	0.042
Mild	819/2,298 (35.6)	368/1,077 (34.2)	451/1,221 (36.9)	0.058
Moderate	807/2,298 (35.1)	391/1,077 (36.3)	416/1,221 (34.1)	0.047
Severe	599/2,298 (26.1)	288/1,077 (26.7)	311/1,221 (25.5)	0.029
TAPSE, mm	18 [15-21] (n=2,208)	18 [15-21] (n=1,080)	18 [15-21] (n=1,128)	0.059
PASP, mmHg	45 [35-55] (n=2,291)	46 [36-57] (n=1,125)	43 [33-54] (n=1,166)	**0.198**

Values are n/N (%) or median (Q1-Q3) (n). **Bold** values indicate SMD >0.1. *3D=three-dimensional; EROA=effective regurgitant orifice area; LAVi=left atrial volume index; LVEF=left ventricular ejection fraction; LVEDD=left ventricular end diastolic diameter; LVESD=left ventricular end systolic diameter; LVEDVi=left ventricular end diastolic volume index; MR=mitral regurgitation; MMR=mixed mitral regurgitation; MPG=mean transmitral pressure gradient; MVOA=mitral valve orifice area; PASP=pulmonary artery systolic pressure; PMR=primary MR; SMD=standardized mean difference; SMR=secondary MR; TAPSE=tricuspid annular plane systolic excursion; TR=tricuspid regurgitation; VC=vena contracta.*

Supplemental Table 3. MVARC-Defined Technical Success According to Baseline 3D-MVOA

	Overall (N=1,189)	MVOA <4 cm^2 (N=338)	MVOA ≥4 cm^2 (N=851)	*p*-value
Absence of procedural mortality	1,188/1,189 (99.9)	337/338 (99.7)	851/851 (100)	0.284
Successful access, delivery, and retrieval of the device delivery system	1,181/1,189 (99.3)	335/338 (99.1)	846/851 (99.4)	0.695
Successful deployment and correct positioning of the first intended device	1,168/1,189 (98.2)	327/338 (96.7)	841/851 (98.8)	**0.027**
Freedom from emergency surgery or reintervention related to the device or access procedure	1,186/1,189 (99.7)	338/338 (100)	848/851 (99.6)	0.563
Values are n/N (%). **Bold** values indicate statistical significance. *3D=three-dimensional*; *MVARC=mitral valve academic research consortium; MVOA=mitral valve orifice area*				

Supplemental Table 4. Narratives for MVARC-Defined Technical Failure According to Baseline 3D-MVOA

**Baseline 3D-MVOA <4 cm²**	
#1	The patient was intended to be treated with a PASCAL P10. Repeated clasping attempts resulted in persistent residual MR, prompting a switch to PASCAL Ace. After multiple further attempts, a worsening of MR was observed, leading the operators to switch to a MitraClip XT. As the first intended device was not implanted, the procedure did not meet criteria for technical success.
#2	The patient was intended to be treated with a PASCAL P10. Repeated clasping attempts resulted in persistent residual MR, and implantation of a second device would not have been feasible due to the small MVOA. A switch to PASCAL Ace was associated with further worsening of MR, prompting a return to a PASCAL P10. As the first intended device was not implanted, the procedure did not meet criteria for technical success.
#3	The patient was intended to be treated with a PASCAL Ace. After central clasping, a marked increase in the transmitral gradient (10.5 mmHg) was observed, indicating hemodynamically relevant mitral stenosis. The device was therefore retrieved, and the procedure was aborted without implantation of the first intended device.
#4	The patient was intended to be treated with a PASCAL Ace. Central clasping resulted in a marked increase in the transmitral gradient (>9 mmHg), indicating hemodynamically relevant mitral stenosis. The device was therefore retrieved, and the procedure was aborted without implantation of the first intended device.
#5	The patient was intended to be treated with a PASCAL Ace. Although initial clasping resulted in MR reduction, the residual broad jet could only be split and implantation of a second device was not feasible due to limited MVOA. Owing to pronounced leaflet tethering, the operators switched to a PASCAL P10, which was ultimately implanted, achieving relevant MR reduction. As the first intended device was not implanted, the procedure did not meet criteria for technical success.
#6	The patient was intended to be treated with a PASCAL Ace. Owing to a very commissural flail segment, correct device positioning could not be achieved despite multiple clasping attempts with different angulations. Although a PASCAL Ace was ultimately implanted in a lateral position, the flail segment could not be captured and residual MR remained severe. Implantation of a second device was not feasible due to the limited MVOA.
#7	The patient was intended to be treated with a PASCAL Ace. Despite acceptable MR reduction after multiple clasping attempts, an unacceptably elevated transmitral gradient (6.7 mmHg) with a reduced MVOA was observed. The device was therefore retrieved and not implanted.
#8	The patient was intended to be treated with a PASCAL Ace. Central positioning resulted in a marked increase in transmitral gradient (7 mmHg) and a critically reduced MVOA. Despite some MR reduction, the device could not be left in place because of hemodynamically relevant mitral stenosis and was therefore retrieved.

#9	The patient was intended to be treated with a PASCAL P10. Due to severe calcification of the mitral valve leaflets, correct device positioning and effective MR reduction could not be achieved, and the procedure was terminated without device implantation.
#10	The patient was intended to be treated with a PASCAL P10. Despite attempts at medial, lateral, and central positioning, no meaningful procedural result could be achieved. The intervention was therefore aborted, and the patient was referred for surgical mitral valve repair without device implantation.
#11	The intended device is not described. During the procedure, partial leaflet detachment occurred, therefore the procedure was regarded as technical failure.
#12	The patient was intended to be treated with a PASCAL Ace. During the procedure, partial device detachment occurred; the device was retrieved, and the intervention was aborted without successful device implantation.
#13	The patient was intended to be treated with a PASCAL Ace. Device implantation resulted in significant mitral stenosis, necessitating a change in device strategy. The procedure was subsequently completed with implantation of a PASCAL P10.
#14	The procedure was aborted due to insufficient echocardiographic visualization, and no device was implanted.
**Baseline 3D-MVOA ≥4 cm^2**	
#1	The patient was intended to be treated with a PASCAL P10. Attempts to address central, medial, and lateral MR jets were unsuccessful and associated with worsening of MR. Further clasping attempts were limited by poor echocardiographic visualization and an unfavorable posterior trajectory, and a defect of the device steering mechanism was identified. The procedure was therefore aborted.
#2	The patient was intended to be treated with a PASCAL P10. During the procedure, single leaflet device attachment occurred, resulting in technical failure.
#3	The patient was intended to be treated with a PASCAL P10. Repeated clasping attempts did not result in relevant MR reduction because the PML could not be stably captured and was repeatedly displaced by chordae. A switch to PASCAL Ace was therefore performed; although secure medial grasping was ultimately achieved and MR was reduced, a residual lateral jet persisted. The procedure was completed with implantation of a PASCAL Ace rather than the first intended device.
#4	The patient was intended to be treated with a PASCAL P10. Multiple clasping attempts failed to achieve effective MR reduction because the restrictive posterior mitral leaflet could not be securely captured due to chordal interference, and the device was not implanted. The operators therefore switched to a PASCAL Ace, which was implanted after two grasping attempts, resulting in relevant MR reduction with residual bilateral jets.
#5	The patient was intended to be treated with a PASCAL Ace. Despite multiple clasping attempts in a central position, a residual flail segment persisted adjacent to the device. The operators therefore switched to a PASCAL P10, which was successfully implanted after central clasping with independent anterior leaflet capture, resulting in marked MR reduction.
#6	The patient was intended to be treated with a PASCAL Ace. After two clasping attempts in a centromedial position, relevant residual MR persisted with an elevated

	transmitral gradient, and implantation of a second device was not feasible. The device was therefore retrieved, and a PASCAL P10 was implanted after a single centromedial grasp with simultaneous leaflet capture, resulting in relevant MR reduction.
#7	The patient was intended to be treated with a PASCAL Ace. Despite multiple clasping attempts, no effective MR reduction could be achieved because of inadequate echocardiographic visualization, which prevented reliable assessment of device insertion, angulation, and positioning. In addition, an unfavorable transmitral gradient after clasping precluded a multi-device strategy. The procedure was therefore aborted.
#8	The patient was intended to be treated with a PASCAL Ace and PASCAL P10. During the procedure, the lateral clip detached from the posterior mitral leaflet, necessitating surgical mitral valve replacement.
#9	The patient was intended to be treated with a PASCAL P10. Correct device positioning could not be achieved and the procedure was aborted.
#10	The procedure was aborted due to persistently high mean transmitral gradients despite multiple repositioning attempts, and no PASCAL device was implanted.
#11	The procedure was aborted due to pronounced iliac vessel kinking, which precluded transseptal access, and no device was implanted.
#12	The reason for aborting the procedure was not reported, nor were the intended device(s).
#13	Despite multiple changes in device positioning and use of both PASCAL P10 and ACE, regurgitant volume and left atrial pressure increased, leading to termination of the procedure without successful device implantation.
#14	The procedure was aborted due to cardiac arrest requiring cardiopulmonary resuscitation and initiation of venoarterial extracorporeal membrane oxygenation.
#15	The patient was intended to be treated with a PASCAL Ace. The procedure was terminated due to suboptimal MR reduction in combination with relevant mitral stenosis, indicated by a mean transmitral gradient >5 mmHg.
3D=three-dimensional; MR=mitral regurgitation; MVOA=mitral valve orifice area; PML=posterior mitral leaflet	

Supplemental Table 5. Device Distribution According to Baseline 3D-MVOA in Patients Treated After PASCAL ACE Introduction

	**Overall (N=891)**	**MVOA <4 cm^2 (N=276)**	**MVOA ≥4 cm^2 (N=615)**	***p*-value**
**Device type**				**<0.001**
No device	10/891 (1.1)	6/276 (2.2)	4/615 (0.7)	0.078
P10 only	132/891 (14.8)	53/276 (19.2)	79/615 (12.8)	**0.018**
Ace only	711/891 (79.8)	215/276 (77.9)	496/615 (80.7)	0.392
Both P10 and Ace	38/891 (4.3)	2/276 (0.7)	36/615 (5.9)	**<0.001**

Values are n/N (%). **Bold** values indicate statistical significance. *3D=three-dimensional; MVOA=mitral valve orifice area*

Supplemental Table 6. Sensitivity Analyses of Main Outcomes Stratified by MR Etiology

	**MVOA <4 cm^2**	**MVOA ≥4 cm^2**	***p*-value**	**Adjusted OR (95% CI), *p***
**Primary MR**				
Technical success	118/122 (96.7)	291/293 (99.3)	0.064	0.19 (0.03–1.09), 0.062
MR ≤1+ at discharge	85/120 (70.8)	206/287 (71.8)	0.943	0.92 (0.56–1.51), 0.738
**Secondary MR**				
Technical success	114/120 (95.0)	404/414 (97.6)	0.247	0.44 (0.15–1.29), 0.133
MR ≤1+ at discharge	81/115 (70.4)	290/407 (71.3)	0.957	0.87 (0.54–1.41), 0.573
**Mixed MR**				
Technical success	91/95 (95.8)	141/144 (97.9)	0.440	0.25 (0.04–1.77), 0.165
MR ≤1+ at discharge	66/89 (74.2)	104/142 (73.2)	0.999	0.98 (0.51–1.86), 0.944

Values are n/N (%) or adjusted ORs, which are from mixed-effects logistic regression with random intercept for center; reference category is 3D-MVOA ≥cutoff. **Bold** values indicate statistical significance. *MR=mitral regurgitation; MVOA = mitral valve orifice area*

Supplemental Table 7. Sensitivity Analyses of Main Outcomes Across 3D-MVOA Thresholds

	**MVOA <cutoff**	**MVOA ≥cutoff**	***p*-value**	**Adjusted OR (95% CI), *p***
**Threshold <4.0 cm^2**				
Technical success	324/338 (95.9)	836/851 (98.2)	**0.028**	0.33 (0.15–0.71), **0.005**
MR ≤1+ at discharge	233/325 (71.7)	600/836 (71.8)	>0.999	0.89 (0.66–1.21), 0.462
**Threshold <3.5 cm^2**				
Technical success	179/184 (97.3)	981/1,005 (97.6)	0.995	0.61 (0.22–1.71), 0.349
MR ≤1+ at discharge	131/177 (74.0)	702/984 (71.3)	0.525	0.93 (0.63–1.37), 0.720
**Threshold <3.0 cm^2**				
Technical success	76/78 (97.4)	1084/1,111 (97.6)	0.715	0.66 (0.15–2.97), 0.586
MR ≤1+ at discharge	53/75 (70.7)	780/1086 (71.8)	0.934	0.73 (0.42–1.26), 0.262

Values are n/N (%) or adjusted ORs, which are from mixed-effects logistic regression with random intercept for center; reference category is 3D-MVOA ≥cutoff. **Bold** values indicate statistical significance. *MR=mitral regurgitation; MVOA = mitral valve orifice area*

Supplemental Table 8. Era-Adjusted Analysis of Main Outcomes (3D-MVOA <4.0 vs. ≥4 cm^2)

	**Adjusted OR (95% CI, *p***
Technical success	0.32 (0.15–0.70), **0.004**
MR ≤1+ at discharge	0.87 (0.64–1.18), 0.380

Values are odds ratios with 95% confidence intervals from mixed-effects logistic regression with random intercept for center, additionally adjusted for PASCAL device era (P10-only, P10+Ace, Precision). Reference category is 3D-MVOA ≥4.0 cm^2. **Bold** values indicate statistical significance. *MR=mitral regurgitation; MVOA=mitral valve orifice area*
