# Supplementary material for: Small Mitral Valve Orifice Area in Mitral Valve Transcatheter Edge-to-Edge Repair: The REPAIR Study
Source: JACC Adv. 2026 Jun 25;5(8):103014. doi: 10.1016/j.jacadv.2026.103014 (PMC13531619; doi:10.1016/j.jacadv.2026.103014)
Supplement: Supplementary file 2 [file mmc2.docx]

† **Pubmed investigator list**

Ralph-Stephan von Bardeleben, MD^q^

Cecilia Ennin, cand. med.^k,l^

Kai Peter Friedrichs, MD^e,f^

Christina Grothusen, MD^n^

Malte Kelm, MD^c,h^

Georg Nickenig, MD^c,p^

Kerstin Piayda, MD^i^

Amin Polzin, MD^c,h^

Tobias Ruf, MD^q^

Leonhard Moritz Schneider, MD^j^

Holger Thiele, MD^g^

Fabian Voss, MD^c,h^

Laila Widmann, MD^i^
